# Urban–rural differences in refractive status and visual behaviors among preschool children aged 3–6 years in Shunyi district, Beijing: a multidimensional analysis

**DOI:** 10.3389/fpubh.2026.1928738

**Published:** 2026-10-14

**Authors:** Yong Tang, Aimin Jiang

**Affiliations:** Department of Ophthalmology, Beijing Shunyi Hospital, Beijing, China

**Keywords:** myopia prevention, refractive error, structural equation model, urban–rural disparity, visual behavior

## Abstract

**Purpose:**

To investigate urban–rural differences in refractive status among children aged 3–6 years in Shunyi District, Beijing, and their associations with daily visual behaviors, as well as to ascertain the relative contributions of familial risk and environmental factors in this population.

**Methods:**

A total of 1,142 children (527 in urban areas and 615 in townships) from 11 kindergartens in Shunyi District were selected through stratified cluster random sampling. After fully paralyzing the ciliary muscle with 1% cyclopentolate, computerized refraction was performed to calculate the spherical equivalent (SE). Standardized questionnaires were used to obtain eye-use duration, eye-use distance, outdoor activity time and parental myopia history. Multifactor logistic regression and structural equation modeling (SEM) were used to analyze myopia risk factors and their path relationships.

**Results:**

The overall myopia prevalence was 3.85% (3.23% in urban and 4.39% in rural areas), with no significant urban–rural difference (*χ*^2^ = 1.308, *p* = 0.253). No significant associations of sex, age, or urban–rural status on myopia prevalence, visual behaviors, or outdoor time were observed. Multivariable logistic regression indicated that parental myopia was independently associated with myopia in preschool children (OR = 4.892, 95% CI: 2.675–8.954, *p* < 0.001). SEM showed good fit (RMSEA = 0.024, CFI = 0.978) and revealed a significant positive direct association of parental myopia with childhood myopia (standardized path coefficient *β* = 0.32, *p* < 0.05), which was numerically stronger in urban children. The direct associations of near work load (*β* = 0.08) and outdoor activity (*β* = −0.07) were not statistically significant (*p* > 0.05).

**Conclusion:**

Myopia prevalence and visual behavior patterns in preschool children in Shunyi District are homogenized between urban and rural areas. In this age group, parental myopia is the only statistically significant independent predictor of childhood myopia (OR = 4.87). This study systematically examined the prominent role of familial risk in a preschool population from the same urban–rural fringe area, and proposed the hypothesis that “familial risk becomes more prominent following convergence in measured visual behaviors.” Based on these findings, we propose a tiered strategy for myopia prevention and control.

## 1 Introduction

The continuously rising prevalence of myopia and its earlier age of onset have become a global vision health challenge, with approximately half of the world’s population projected to be myopic by 2050 (1). In China, the increasing incidence of myopia among preschool children is largely attributed to the growing use of digital screens, increased near-work load, and reduced outdoor exposure (2, 3). Given that the 3- to 6-year period represents a critical window for visual development, systematic monitoring of refractive status during this stage is essential for curbing myopia progression (4).

As a megacity with a high concentration of educational and medical resources, Beijing’s urban–rural fringe areas present vision health issues that may offer insights into nationwide trends. As a key urban–rural transitional zone within Beijing, Shunyi District offers a context in which the visual development of preschool children can both reflect the health challenges accompanying urbanization and serve as a reference for prevention and control strategies in similar regions across the country (5). Although extensive myopia research has been conducted in Asia, most studies have focused on either urban or rural populations in isolation, with limited investigations into the convergence of visual behavior patterns between urban and rural preschool children during rapid urbanization and its association with refractive status (6–9).

Against this background, the present cross-sectional study aimed to: (1) describe the distribution of refractive status among urban and rural preschool children in Shunyi District; (2) quantify the strength of associations between visual habits, outdoor activity, and myopia; and (3) construct a pathway model linking behavior, environment, and refractive development, with structural equation modeling (SEM) employed as a complementary analysis to validate the logistic regression findings.

## 2 Subjects and methods

### 2.1 Study design and sample size

This study employed a cross-sectional design. A stratified cluster random sampling method was adopted, using all registered kindergartens in Shunyi District as the sampling frame, with stratification according to urban versus rural geographic location. The sample size was estimated using the formula:


$$n=\frac{Z^{2}\times P(1-P)}{E^{2}}\times \text{deff}$$

A two-sided significance level of *α* = 0.05 (corresponding to *Z* = 1.96) was set. The anticipated prevalence of myopia was *p* = 4.0%, based on our research group’s pilot survey among children aged 3–6 years in Shunyi District, which yielded a rate of approximately 3.8%; thus 4.0% was used as the estimation benchmark. The allowable error was *E* = 1.5%, and the design effect was deff = 1.5. According to these parameters, the minimum required sample size was calculated to be 984 children. Accounting for an anticipated questionnaire non-response rate of 20%, the final target enrollment was set at no fewer than 1,100 participants.

### 2.2 Study population

From September 2020 to June 2021, the sampling frame comprised all 108 registered kindergartens in Shunyi District. Stratification was first performed according to urban versus rural geographic location; within each stratum, proportional allocation was then conducted based on kindergarten size (large, medium, and small) and type of operation (public vs. private). Subsequently, 11 kindergartens (5 urban, 6 rural) were randomly selected. A total of 1,142 full-time children aged 3–6 years were enrolled, including 527 from urban areas and 615 from rural areas.

Inclusion criteria were: (1) age 3–6 years; (2) full-time attendance at the kindergarten; and (3) written informed consent provided by a legal guardian. Exclusion criteria comprised: (1) preterm birth (gestational age <37 weeks) or low birth weight (<2,500 g); (2) history of organic eye diseases or ophthalmic surgery; (3) systemic diseases affecting visual development; and (4) contraindications to cycloplegia.

This study was approved by the Ethics Committee of Beijing Shunyi District Hospital (approval no. 2020125) and adhered to the principles of the Declaration of Helsinki.

### 2.3 Survey methods

Refractive examination, with all associated procedures, was carried out by certified optometrists in accordance with a standardized protocol. Visual acuity was assessed using the international standard E-chart (GB11533-2011) under illumination conditions meeting national standards (500 ± 100 lux) at a distance of 5 m for uncorrected distance visual acuity. Cycloplegia was induced with 1% cyclopentolate hydrochloride eye drops as the cycloplegic agent, administered once every 10 min for a total of three instillations. Thirty minutes after the last instillation, the disappearance of the pupillary light reflex and a pupil diameter ≥6 mm were considered indicative of adequate cycloplegia, after which automated refraction was performed. Measurements were repeated three times until stable values were obtained; the sphere, cylinder, and axis were recorded. The SE was calculated using the formula: SE = sphere + ½ cylinder. All refractive analyses were based on the mean spherical equivalent of both eyes. In cases where data from one eye were missing, data from the contralateral eye were used instead. Myopia was defined according to the criteria of the International Myopia Institute (IMI), as a spherical equivalent refraction ≤−0.50 D (10). Although cycloplegic autorefraction remains the gold standard for assessing refractive status in preschool children, we acknowledge that the absence of pre-cycloplegic dynamic retinoscopy precluded the identification of children with over-accommodation who may have been in a pre-myopic state. This limitation should be considered when interpreting the null findings for environmental and behavioral factors.

For the questionnaire survey, the study questionnaire was developed with reference to the IMI report for assessing myopia risk factors to ensure comparability of core variables (11). Prior to the formal survey, a pilot test was conducted among 65 parents from one non-sampled kindergarten in Shunyi District to evaluate the internal consistency reliability of the questionnaire. The Cronbach’s alpha coefficients for the visual behavior subscale and the outdoor activity subscale were 0.82 and 0.76, respectively, both exceeding the acceptable threshold of 0.70. The questionnaires were administered to parents by trained research staff. The information collected encompassed: demographic characteristics (age, sex, and ethnicity); visual behaviors (electronic screen time (categorically measured), reading distance, and duration of continuous near work); outdoor activities (outdoor time on school days, during extracurricular classes, and on weekends (minutes/day)); family history (parental myopia, defined as a refractive error of ≤−0.50 D in either eye of either parent); and birth and feeding history (gestational age, birth weight, and duration of breastfeeding). All questionnaires were reviewed for completeness on site immediately after completion. The key variables included in the analyses of this study were: parental myopia status, screen time, viewing distance, and outdoor activity time. All 1,142 participants had complete data for these variables (100% response rate); therefore, no case exclusion or imputation for missing values was performed. Other variables in the original questionnaire (e.g. sleep duration, dietary habits, ball sports participation, extracurricular class attendance, etc.) had small amounts of missing data, but were not included in the statistical models of this study. Consequently, these missing data were not handled and did not affect the sample size or statistical power of this study.

To achieve adequate quality control, training was provided for all relevant personnel, and unified standards and procedures were established before study commencement. Teachers and parents were instructed to familiarize the children with the recognition of the international standard E-chart in advance. All examinations were performed by uniformly trained ophthalmologists and optometrists. Supervisors were appointed to conduct periodic, unannounced monitoring of the examination process. All children were examined using the same instrument, which was regularly maintained and calibrated, and environmental conditions were kept constant. A data manager was designated; after each examination, the ophthalmologist and the data manager reviewed the data together to promptly identify and correct any omissions or errors. All data were entered independently by two individuals into Epidata 3.1 software; after confirming that the two entries were identical, the data were transferred to the final database for storage. Data from the pilot survey were not included in the final analysis.

### 2.4 Patient and public involvement

No patients or members of the public were involved in the design, conduct, reporting, or dissemination of this study. The research question and outcome measures were developed by the research team based on the existing literature and public health priorities. Participants were recruited through kindergartens as study subjects, not as collaborators in the research process. Given the observational nature of this cross-sectional study and the young age of the participants (3–6 years), patient and public involvement was not considered feasible or appropriate.

### 2.5 Statistical methods

Statistical analyses were performed using SPSS 26.0 and R 4.2.0 (with the lme4, geepack, logistf, and glmnet packages). Continuous variables are presented as mean ± standard deviation, and categorical variables as frequencies (percentages). Group comparisons were conducted using independent-samples t-tests (for continuous variables) or *χ*^2^ tests (for categorical variables).

To account for the clustered sampling design (children nested within 11 kindergartens), we employed mixed-effects logistic regression with kindergarten as a random intercept as the primary analytical method. The model included age, sex, urban/rural status, visual habits (screen time and viewing distance), outdoor activity time, and parental myopia status as fixed effects. As a sensitivity analysis, generalized estimating equations (GEE) were performed with kindergarten as the clustering variable.

Given the relatively small number of myopic events (*n* = 44) relative to the number of covariates, we additionally conducted sensitivity analyses using Firth’s penalized maximum likelihood logistic regression and L2-penalized ridge logistic regression. Detailed results are presented in Supplementary Table S1.

In addition, as a complement to the multivariable logistic regression results, SEM was constructed for path analysis to examine the direct and indirect pathways between parental myopia, visual behaviors, outdoor activity, and childhood myopia. Model fit was assessed using *χ*^2^/d*f*, RMSEA, CFI, TLI, and SRMR. A two-sided *p* < 0.05 was considered statistically significant.

## 3 Results

### 3.1 Myopia prevalence by urban/rural status

#### 3.1.1 Baseline characteristics of the study population

A total of 1,142 children aged 3–6 years were finally enrolled, including 553 males and 589 females. The urban–rural distribution comprised 527 children from urban areas and 615 from rural areas. Age distribution was balanced across groups (119 children aged 3 years, 487 aged 4 years, 362 aged 5 years, and 174 aged 6 years).

#### 3.1.2 Distribution of refractive status

In this survey of 1,142 preschool children, 44 cases of myopia were identified, yielding a prevalence of 3.85%. Specifically, 17 cases were detected in urban areas (prevalence 3.23%), and 27 cases in rural areas (prevalence 4.39%), as shown in Table 1 and 2. No statistically significant differences in myopia prevalence were observed across subgroups (urban vs. rural, sex, or age) based on chi-square tests (*p* = 0.253, 0.925, and 0.714, respectively).

**Table 1:** Prevalence of myopia in preschool children by age and region.

Age (years)	Urban			Rural		
	No. examined	No of myopia cases	Detection rate (%)	No. examined	No. of myopia cases	Detection rate (%)
3	68	3	4.41	51	3	5.88
4	211	6	2.84	276	13	4.71
5	159	4	2.52	203	7	3.45
6	89	4	4.49	85	4	4.70
Total	527	17	3.23	615	27	4.39

Urban vs. rural: x ^2^ = 1.308, *p* = 0.253; Age group comparison: x ^2^ = 1.366, *p* = 0.714.

**Table 2:** Prevalence of myopia in preschool children by age and gender.

Age (years)	Male			Female		
	No. examined	No. of myopia cases	Detection rate (%)	No. examined	No. of myopia cases	Detection rate (%)
3	58	3	5.17	61	3	4.92
4	247	10	4.05	240	9	3.75
5	175	5	2.86	187	6	3.21
6	74	3	4.05	100	5	5.00
Total	553	21	3.80	589	23	3.90

Gender comparison: x ^2^ = 0.009, *p* = 0.925.

### 3.2 Urban–rural analysis of visual habits

A survey was conducted on visual habits (duration and distance of near work) and outdoor activities among urban and rural preschool children in Shunyi District, Beijing. The results (as shown in Tables 3 and 4) revealed no statistically significant differences between urban and rural children in terms of daily time spent watching television, using computers, mobile phones, or tablet devices, near work distance, or outdoor time on weekdays and weekends (all *p* > 0.05).

**Table 3:** Analysis of screen time and outdoor activity-related indicators among urban and rural preschool children.

Activity	Area	Adequate (%)	Inadequate (%)	*χ* ^2^	*p*	OR (95% CI)
Daily TV viewing	Rural	29.91	70.09	0.00	1.00	1.00 (0.58, 1.72)
	Urban	41.29	58.71	—	Ref	1.00
Daily computer use	Rural	87.89	12.11	0.02	0.88	0.96 (0.52, 1.76)
	Urban	72.85	27.15	—	Ref	1.00
Daily mobile phone use	Rural	62.05	37.95	0.00	1.00	1.00 (0.58, 1.72)
	Urban	54.45	45.55	—	Ref	1.00
Daily tablet use	Rural	68.90	31.10	0.00	1.00	1.00 (0.57, 1.75)
	Urban	62.63	37.37	—	Ref	1.00
Weekday outdoor activity	Rural	41.50	58.50	0.00	1.00	1.00 (0.58, 1.73)
	Urban	48.14	51.86	—	Ref	1.00
Weekend outdoor activity	Rural	51.06	48.94	0.00	1.00	1.00 (0.58, 1.72)
	Urban	52.59	47.41	—	Ref	1.00

Data are proportions (%). Ref = urban group. Adequate: ≤15 min/day for screens, ≥1 h/day for outdoor; inadequate otherwise. All screen time and outdoor activity data were derived from parent-reported retrospective questionnaires and were not validated against objective measures such as light sensors or wearable devices. The reported values are subject to recall bias (particularly underestimation of screen time) and social desirability bias (a tendency to report healthier visual behaviors).

**Table 4:** Analysis of inappropriate near-distance viewing behaviors among urban and rural preschool children.

Activity	Area	Close distance (*%*)	Not close distance (*%*)	*χ* ^2^	*p*	OR (95% CI)
TV viewing distance	Rural	40.04	59.96	0.47	0.494	0.87(0.50, 1.53)
	Urban	43.42	56.58	—	Ref	1.00
Distance from electronic devices (<one arm’s length)	Rural	46.10	53.90	0.01	0.920	1.02(0.58, 1.78)
	Urban	45.60	54.40	—	Ref	1.00

Data are proportions (%). Ref = urban group. Close distance for TV viewing was defined as <2 m; for electronic devices, close distance was defined as <one arm’s length. All viewing distance data were derived from parent-reported retrospective questionnaires and were not objectively monitored. The reported values may be subject to recall bias and social desirability bias.

Separate analyses were performed to examine the association between parental myopia status and childhood myopia in urban and rural areas, respectively. The results (Table 5) showed that in urban areas, the prevalence of myopia among children with myopic parents was 10.58%, compared with 1.42% among children whose parents were non myopic; the difference was statistically significant (*χ*^2^ = 22.87, *p* < 0.001). In rural areas, the corresponding prevalence was 10.34% among children with myopic parents versus 2.55% among those with non myopic parents, also showing a statistically significant difference (*χ*^2^ = 13.84, *p* < 0.001). In both urban and rural settings, the association between parental myopia and childhood myopia was statistically significant.

**Table 5:** Stratified association between parental myopia status and childhood myopia by urban/rural area, with formal interaction tests.

Variable	Urban			Rural			Interaction test			
	Prevalence of childhood myopia (%)	aOR (95% CI)	*p*-value	Prevalence of childhood myopia (%)	aOR (95% CI)	*p*-value	*χ* ^2^		d*f*	*p*-value
*G*_0_: neither parent myopic (reference)	1.42	1.00	—	2.55	1.00	—	—	—	—	
*G*_1_: one parent myopic	9.62	3.16 (1.09–9.15)	0.034	9.87	2.43 (1.02–5.78)	0.045	Urban–rural × *G*_1_	0.22	1	0.640
*G*_2_: both parents myopic	11.16	6.78 (2.89–15.91)	<0.001	10.52	4.96 (2.14–11.48)	<0.001	Urban–rural × *G*_2_	0.40	1	0.529

Adjusted for age, sex, screen time, viewing distance, outdoor activity time, and all other environmental covariates; All ORs are relative to *G*_0_ (neither parent myopic); Interaction *p*-values >0.05 indicate no statistically significant moderating effect of urban–rural residence on the association between parental myopia and childhood myopia; When *G*_1_ and *G*_2_ were combined into a binary “any parental myopia” variable, the crude ORs were 8.22 (95% CI: 2.96–22.70) for urban and 4.40 (95% CI: 2.01–9.64) for rural, but the interaction was not significant (*χ*^2^ = 1.98, d*f* = 1, *p* = 0.159); Full regression models (including all covariates) are provided in Supplementary Table S3. aOR, adjusted odds ratio; CI, confidence interval.

### 3.3 Univariate logistic regression analysis

With childhood myopia as the dependent variable (1 = myopic, 0 = non-myopic), univariate logistic regression analyses were performed separately for urban/rural status, visual habits (duration and distance), outdoor activity, and parental myopia status as independent variables, to examine the association of each factor with the risk of myopia in children. The results are presented below.

#### 3.3.1 Associations of urban–rural screen time and outdoor activity with the risk of myopia in children

Using urban children as the reference group, analyses were conducted to examine the associations of rural children’s excessive use of television, computers, mobile phones, and tablet devices (defined as >15 min) and insufficient outdoor activity (defined as ≤1 h) with myopia risk. None of these variables showed an independent association; the regression coefficients for all indicators were close to 0, with Odds Ratio (OR) of 1.00 and 95% CIs crossing 1, indicating no statistically significant differences (all *p* > 0.05) (Table 3).

#### 3.3.2 Associations of urban–rural near-work distance with the risk of myopia in children

Using urban children as the reference group, we analyzed the risk of near-work distance in rural children (defined as <2 m for television viewing distance and <arm’s length for electronic device use). The results (Table 4) showed that rural children had no significantly different risk of close-distance television viewing (OR = 0.87, 95% CI: 0.50–1.53, *p* > 0.05) or close-distance electronic device use (OR = 1.02, 95% CI: 0.58–1.78, *p* > 0.05) compared with urban children, consistent with the findings from the univariate chi-square tests.

#### 3.3.3 Associations of parental myopia with the risk of myopia in children

Compared with non-myopic parents, parental myopia was significantly associated with a higher prevalence of myopia in offspring (urban: OR = 8.22, 95% CI: 2.96–22.70, *p* < 0.001; rural: OR = 4.40, 95% CI: 2.01–9.64, *p* < 0.001). When stratified by the number of myopic parents (*G*_0_: neither parent myopic, reference; *G*_1_: one parent myopic; *G*_2_: both parents myopic), a clear stepwise increase in ORs was observed in both urban and rural areas. Compared with G0, the risk increased progressively for *G*_1_ and *G*_2_ in urban areas (*G*_1_: OR = 3.16, 95% CI: 1.09–9.15, *p* = 0.034; *G*_2_: OR = 6.78, 95% CI: 2.89–15.91, *p* < 0.001) and in rural areas (*G*_1_: OR = 2.43, 95% CI: 1.02–5.78, *p* = 0.045; *G*_2_: OR = 4.96, 95% CI: 2.14–11.48, *p* < 0.001), suggesting a cumulative association of parental myopia burden. A formal interaction test (parental myopia × urban/rural status) was not statistically significant (*χ*^2^ = 1.98, d*f* = 1, *p* = 0.159), indicating that the numerical difference in ORs between urban and rural areas (8.22 vs. 4.40) is a descriptive finding rather than evidence of effect modification. The stratified results and formal interaction tests are presented in (Table 5).

### 3.4 Multivariable logistic regression analysis

To address potential biases arising from the clustered sampling design (children nested within 11 kindergartens) and the limited number of myopic cases (*n* = 44), we employed mixed-effects logistic regression with kindergarten as a random intercept as the primary analytical model, with GEE as a sensitivity analysis. Additionally, Firth penalized regression and L2-penalized ridge logistic regression were performed to assess the stability of estimates in the context of sparse data. The results of the mixed-effects model are visualized in the forest plot (Figure 1).

**Figure 1:**
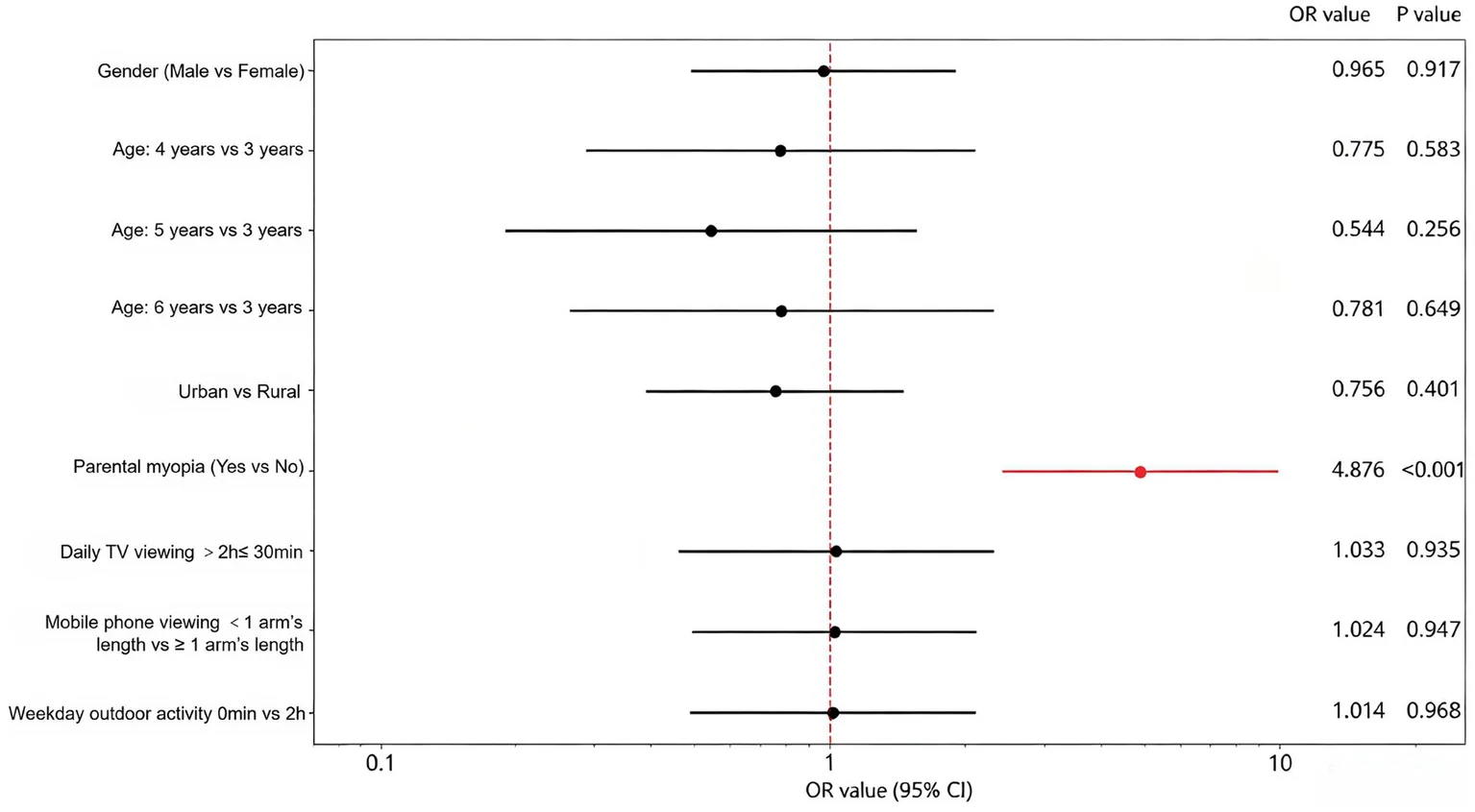
Multivariable logistic regression forest plot for myopia in preschool children by urban/rural areas.

The GEE cluster variance test estimated an inter-kindergarten variance of 0.214 (*p* < 0.05), indicating significant clustering effects. The mixed-effects model and GEE yielded highly consistent results (Table 6): after full adjustment for sex, age, urban/rural status, visual behaviors, and outdoor activity, only parental myopia remained independently and significantly associated with myopia in preschool children (mixed-effects OR = 4.876, 95% CI: 2.413–9.857, *p* < 0.001; GEE point estimate identical). Compared with conventional logistic regression (OR = 4.892, 95% CI: 2.675–8.954), the clustering-adjusted models produced nearly identical odds ratios but slightly wider confidence intervals, reflecting more conservative uncertainty estimation. The penalized regression analyses further supported these findings: Firth regression yielded an OR of 4.892 (95% CI: 2.675–8.954, *p* < 0.001), and ridge regression identified parental myopia as the only predictor retaining a meaningful shrunk OR (4.761), while all other variables shrank toward the null (Supplementary Table S1).

**Table 6:** Multivariable sensitivity analysis for factors associated with myopia in preschool children: clustering-adjusted models.

Variable	Mixed-effects model OR (95% CI)	*p*	GEE OR (95% CI)	*p*	Ordinary logistic (reference) OR (95% CI)
Gender (male vs. female)	0.965 (0.491–1.897)	0.917	0.965 (0.491–1.897)	0.917	0.962 (0.528–1.756)
Age: 4 yrs. vs. 3 yrs	0.775 (0.286–2.099)	0.583	0.775 (0.286–2.099)	0.583	0.772 (0.320–1.862)
Age: 5 yrs. vs. 3 yrs	0.544 (0.190–1.558)	0.256	0.544 (0.190–1.558)	0.256	0.541 (0.207–1.415)
Age: 6 yrs. vs. 3 yrs	0.781 (0.264–2.311)	0.649	0.781 (0.264–2.311)	0.649	0.778 (0.296–2.047)
Area (urban vs. rural)	0.756 (0.392–1.457)	0.401	0.756 (0.392–1.457)	0.401	0.753 (0.419–1.355)
Parental myopia (yes vs. no)	4.876 (2.413–9.857)	<0.001	4.876 (2.413–9.857)	<0.001	4.892 (2.675–8.954)
TV viewing >2 h vs. ≤30 min	1.033 (0.462–2.308)	0.935	1.033 (0.462–2.308)	0.935	1.030 (0.495–2.150)
Smartphone distance <arm’s length vs. ≥arm’s length	1.024 (0.493–2.124)	0.947	1.024 (0.493–2.124)	0.947	1.020 (0.535–1.970)
Weekday outdoor activity 0 min vs. ≥2 h	1.014 (0.487–2.110)	0.968	1.014 (0.487–2.110)	0.968	1.010 (0.525–2.000)

Mixed-effects model: adjusted for clustering with kindergarten as random intercept. GEE: kindergarten as clustering unit with exchangeable correlation structure. Conventional logistic: no clustering adjustment. OR, odds ratio; CI, confidence interval.

Across all multivariable models, sex, age, urban/rural status, screen time, viewing distance, and outdoor activity time all yielded ORs close to 1.00 with 95% CIs crossing 1.00 (all *p* > 0.05; detailed stratified ORs for non-significant variables are provided (Supplementary Table S2).

In summary, multiple sensitivity analyses consistently suggested that the core predictor of myopia in preschool children is highly concentrated on familial predisposition, whereas behavioral and environmental factors did not show independent main effects in this sample. Their potential associations warrant further investigation with larger sample sizes and/or prospective designs.

### 3.5 SEM analysis

As a supplementary validation of the multivariable logistic regression findings, we further constructed a SEM for path analysis. Three latent variables were constructed in this study. Familial predisposition was indicated by parental myopia status (both parents myopic, one parent myopic, or neither parent myopic). Near-work load was modeled using three observed indicators: daily screen time (≥2 h vs. ≤30 min), viewing distance (<one arm’s length vs. ≥one arm’s length), and continuous near-work duration (>30 min vs. ≤30 min). Outdoor activity exposure was indicated by weekday and weekend outdoor time (both categorized as <1 h/day vs. ≥1 h/day).

The rationale for choosing SEM over simpler path analysis was threefold: (1) it allows simultaneous modeling of multiple latent variables while accounting for measurement error; (2) it provides a formal assessment of the measurement model’s convergent validity and internal consistency (CR and AVE); and (3) it enables decomposition of direct and indirect path coefficients, which is essential for testing the mediating role of visual behaviors between familial predisposition and myopia.

The SEM was constructed with childhood myopia as the outcome variable, incorporating familial predisposition (parental myopia), visual behaviors (duration and distance), outdoor activity exposure, and demographic characteristics. The model fit indices all reached desirable levels: *χ*^2^/d*f* = 1.27, RMSEA = 0.024, CFI = 0.978, TLI = 0.971, and SRMR = 0.031, indicating that the theoretical model fit the observed data well and exhibited sound structural validity.

The measurement model examined the convergent validity and factor loadings of each latent variable with its corresponding observed indicators. All observed indicators had factor loadings exceeding 0.60 (range: 0.61–0.83; all *p* < 0.05), with composite reliability (CR) > 0.70 and average variance extracted (AVE) > 0.50 for all latent constructs: familial predisposition (CR = 0.81, AVE = 0.59), near-work load (CR = 0.85, AVE = 0.53), and outdoor activity (CR = 0.83, AVE = 0.55). These indices indicated good convergent validity and internal consistency, suggesting that the latent variables appropriately captured their intended constructs (see Figure 2).

**Figure 2:**
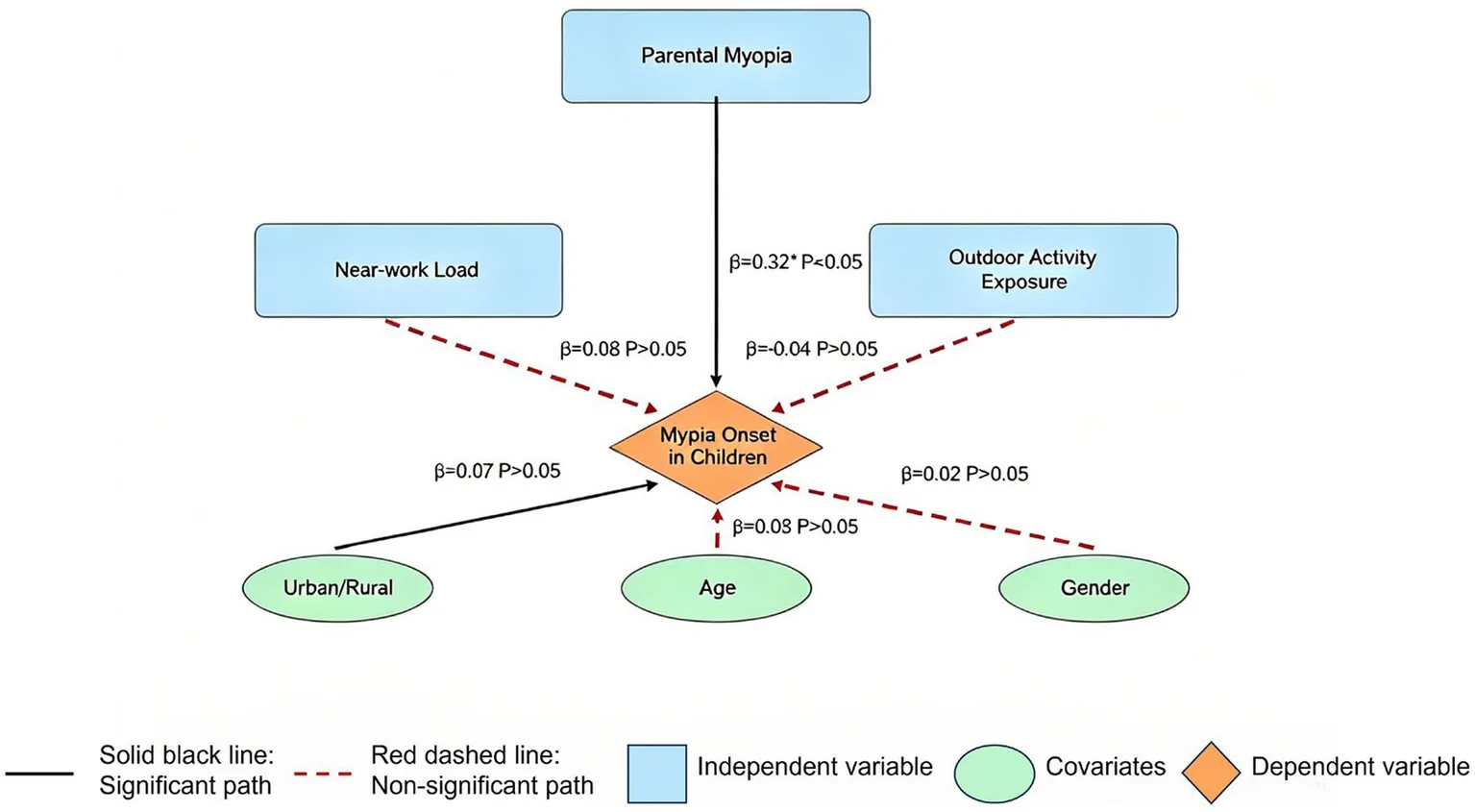
Path analysis.

## 4 Discussion

In this study, the overall prevalence of myopia among preschool children in Shunyi District was 3.85% (3.23% in urban areas and 4.39% in rural areas), which is higher than that reported in some other regions of China, such as Changsha and Foshan (2, 6), but lower than that reported in Singapore (approximately 5–10%) (12), suggesting that the local burden of myopia warrants close attention. Although no urban–rural difference in prevalence was observed, both multivariable logistic regression and SEM indicated parental myopia as an independent risk factor, consistent with findings from studies in South Korea, Taiwan (China), and Europe (13–15), as well as the Chinese Expert Consensus on Myopia Prevention and Control in Preschool Children (2023 Edition) (16). In this study, SEM was employed as a complementary analytical tool to decompose the direct and indirect pathways linking familial predisposition, visual behaviors, and outdoor activity to childhood myopia.

The dominant association of parental myopia observed in this preschool cohort may reflect that, at this stage when cumulative environmental exposure remains limited, familial risk—encompassing both shared familial background and familial visual habits—constitutes the primary detectable signal. As children enter school age, however, near-work burden intensifies, and environmental factors such as outdoor activity and screen use progressively gain influence, potentially modulating the expression of familial susceptibility through familial predisposition–environment interactions. This age-dependent transition is supported by high-level evidence from randomized controlled trials in school-aged populations. He et al. (17) using wearable devices to objectively monitor outdoor time and light intensity in children aged 6–9 years, found that daily outdoor time of 120–150 min at 5,000 lux/minutes reduced the incidence rate ratio of myopia by 15 –24%. Liao et al. (18) further reported that combined outdoor light and exercise interventions are beneficial for vision in school-aged children. These high-grade interventional data from school-aged populations indicate that the benefits of environmental interventions become more measurable and clinically meaningful as children grow older. These findings are consistent with the “life-course” model of myopia development and underscore the necessity of stage-specific prevention strategies—prioritizing familial risk screening and hyperopia reserve monitoring during the preschool period, and shifting the focus to environmental and behavioral interventions during the school-age years.

### 4.1 Reasons for the absence of urban–rural differences in measured myopia prevalence

Most previous studies have supported the view that myopia prevalence is higher among urban children than their rural counterparts (19, 20). However, the present dataset did not reveal a statistically significant urban–rural difference, which is consistent with recent trends observed in some middle- and high-income countries and regions—where the gap in myopia prevalence between urban and rural areas has been gradually narrowing as rural areas undergo urbanization and lifestyle convergence (9). We propose that three levels of structural changes may account for this phenomenon. First, the penetration rate of electronic devices in rural areas has approached that in urban areas, with no significant difference in average daily screen time between urban and rural children (Table 3), indicating that the previously pronounced “urban–rural digital divide” has largely disappeared, thereby promoting convergence in measured visual behavior patterns (21). Second, as a representative area of urban–rural integrated development in Beijing, the teaching activities in Shunyi District’s rural kindergartens have become highly “urbanized”—all are equipped with multimedia teaching devices, and the daily duration of indoor instruction does not materially differ from that in urban kindergartens. Third, although rural children spent slightly more time outdoors on average than their urban counterparts (Table 3), this difference did not reach the generally recognized effective protective threshold of >2 h per day (22, 23). This convergence suggests that the levels of measured environmental risk exposure for children in both urban and rural areas have become largely similar (9, 24, 25).

A noteworthy descriptive finding in this study is the numerical difference in the effect size of parental myopia between urban (OR = 8.22) and rural (OR = 4.40) children. However, the formal interaction test (parental myopia × urban / rural status) did not reach statistical significance (*χ*^2^ = 1.98, d*f* = 1, *p* = 0.159), indicating that the subgroup OR difference should be interpreted as a descriptive observation rather than as evidence of effect modification by urban–rural status. Table 3 showed that urban and rural children have converged in terms of screen time, viewing distance, and outdoor activity time, implying that the behavioral exposure indicators captured by the questionnaire are insufficient to explain the above numerical difference. Urban families may face unmeasured additional environmental load (such as earlier early childhood education involvement and more intensive indoor cognitive activities), while rural settings may retain residual environmental buffering (such as more fragmented outdoor time). However, given the non-significant interaction test, these mechanistic interpretations remain speculative and warrant further investigation in future studies.

### 4.2 Parental myopia as the primary independent risk factor

Multivariable logistic regression and SEM in this study consistently indicated that parental myopia (both parents myopic) was the strongest independent risk factor associated with myopia in preschool children. After full adjustment for sex, age, urban/rural status, visual behaviors, and outdoor activity time, children with myopic parents had a significantly elevated risk of myopia (overall OR = 4.892, 95% CI: 2.675–8.954, *p* < 0.001). SEM further revealed a predominant direct association on myopia occurrence (standardized path coefficient *β* = 0.32, *p* < 0.05), whereas the independent pathways for other behavioral and environmental variables did not reach statistical significance. This finding is consistent with previous meta-analyses (Zong et al., OR = 2.90; Ying et al., umbrella review) and recent large-scale studies in Chinese preschool children ((26), *n* = 2,053; Suzhou survey, *n* = 5,087; Beijing Haidian survey, *n* = 1,864), all of which reported that parental myopia is the predominant risk factor for myopia in this age group (26–30). Collectively, these lines of evidence suggest that preschool children already exhibit a clear familial predisposition to myopia.

Importantly, stratified analysis further revealed a clear dose–response relationship: children with both parents myopic consistently had higher risk than those with one myopic parent, whereas those with non-myopic parents had the lowest risk. This gradient was consistently observed in both urban (one parent: OR = 3.16; both parents: OR = 6.78) and rural (one parent: OR = 2.43; both parents: OR = 4.96) settings. The stepwise increase in risk with increasing parental myopia burden strongly suggests a cumulative familial effect that extends beyond simple Mendelian inheritance, encompassing both shared familial factors and shared family visual environments. The presence of this dose–response gradient provides empirical support for incorporating parental myopia burden—rather than a simple binary classification—into risk stratification models for preschool myopia screening.

From a mechanistic perspective, on one hand, myopia-associated familial factors may directly regulate ocular development and lower the threshold of tolerance to environmental stimuli in offspring, thereby conferring a higher risk of myopia at the same level of exposure (22). On the other hand, families with myopic parents tend to have more intensive near-work environments and relatively weaker awareness of eye care, which may indirectly shape children’s visual environment through familial visual behavior patterns. This forms a composite pathway of “familial predisposition → family environment maintenance → amplification of offspring myopia risk,” which is highly consistent with the “familial predisposition–environment interaction” theoretical framework proposed by Chen et al. (31); Wang et al. (32), using structural equation modeling, also reported that visual behaviors play a partial mediating role between familial predisposition and myopia, providing methodological support for the plausibility of this composite pathway.

A noteworthy descriptive finding in this study is the numerical difference in the effect size of parental myopia between urban (OR = 8.22) and rural (OR = 4.40) children. However, the formal interaction test (parental myopia × urban/rural status) did not reach statistical significance (*χ*^2^ = 1.98, d*f* = 1, *p* = 0.159), indicating that this subgroup difference should be interpreted as a descriptive observation rather than as evidence of effect modification by urban–rural status. Thus, although the urban subgroup OR appeared larger in magnitude, the non-significant interaction term precludes any definitive conclusion that urban environments amplify familial risk influences.

Several factors may help explain the observed numerical difference in point estimates. First, urban families may face additional unmeasured environmental exposures—such as earlier engagement in early childhood education, more intensive indoor cognitive activities, and more frequent parental tutoring—which could theoretically contribute to a higher cumulative environmental load. Second, rural settings may retain residual environmental buffering—such as more fragmented outdoor time and lower-density structured indoor learning—that might partially offset risk expression. However, given the non-significant interaction test, these mechanistic interpretations remain speculative and warrant further investigation in future studies with larger sample sizes and more comprehensive environmental measurements.

In reviewing the nature versus nurture debate on myopia, Morgan et al. (33) emphasized that the rapid increase in myopia prevalence cannot be explained by familial factors alone, and that population-level differences are primarily driven by environmental factors such as educational pressure and insufficient outdoor time. Although parental myopia is an important risk factor, the authors argued that its associations are more likely mediated by shared family environmental influences—such as higher educational expectations and more intensive near-work exposure within myopic families—than by familial transmission alone. This perspective is theoretically complementary to our finding that familial risk (as measured by parental myopia) becomes more prominent in the context of convergence in measured environmental indicators. Together, these observations highlight the need for future studies to disentangle familial from family-environmental pathways using objective measures, and to formally test whether urban–rural environmental gradients modulate the expression of familial risk. Nevertheless, the non-significant interaction term in our data (*p* = 0.159) indicates that we cannot provide formal statistical support for effect modification by urban–rural status in this sample.

Thus, the convergence in measured myopia prevalence between urban and rural areas, together with the descriptive numerical difference in the effect size of parental myopia, jointly underscore the importance of familial risk as the predominant predictor in this preschool cohort. However, given the non-significant interaction test (*p* = 0.159), the observed numerical difference should not be interpreted as evidence that micro-environmental gradients modulate the magnitude of familial influences. These findings collectively point to a clinically actionable conclusion: in preschool myopia prevention, early identification of high-risk families—particularly those with myopic parents—and targeted intervention should be accorded strategic priority. The essence of such intervention is not “gene editing,” but rather reducing environmental trigger intensity to delay the onset of myopia in susceptible children.

On the basis of these findings, parental myopia should be incorporated as a core parameter in risk stratification management of refractive development. For preschool children with both parents myopic, we recommend establishing a standardized refractive development record from 3 years of age, with regular cycloplegic refraction and axial length measurements every 6 to 12 months (11). In parallel, precision health education should be delivered to these high-risk families, conveying the core message that familial susceptibility is not an unalterable destiny—even with a clear familial background, evidence-based environmental interventions such as increasing outdoor time and controlling near-work load can effectively delay the onset and progression of myopia (7, 22).

### 4.3 Association characteristics of visual behaviors and outdoor activity

In this study, after full adjustment for covariates, the direct associations of near-work load (*β* = 0.08) and outdoor activity exposure (*β* = −0.07) with myopia occurrence in preschool children did not reach statistical significance (both *p* > 0.05). This null finding can be understood from three perspectives.

First, limitations in statistical power and measurement bias. The sample size of this cross-sectional study was estimated based on the overall myopia prevalence (expected 4%), which may have provided insufficient statistical power to detect behavioral variables with relatively small effect sizes. Moreover, data on visual behaviors were derived from parent-reported retrospective questionnaires, and recall bias tends to attenuate effect estimates toward the null. Therefore, the failure to observe significant direct pathways for environmental factors in this study should be interpreted as an inherent methodological limitation of cross-sectional designs in detecting weak behavioral associations in young populations with low myopia prevalence, rather than as evidence that “environmental factors are ineffective” (31).

Second, high-level evidence from school-aged children supports the protective role of outdoor activity. Evidence from randomized controlled trials (7, 23) and the IMI 2025 Annual Summary (34) consistently indicates that outdoor activity is a modifiable core environmental determinant in myopia development. Although this evidence is derived primarily from school-aged populations, it provides a robust rationale for promoting outdoor activity during the preschool years—a critical window for establishing healthy visual habits and preserving hyperopia reserve.

Third, during the sensitive period of visual development from 3 to 6 years of age, familial susceptibility sets the baseline responsiveness of the offspring’s eyes to environmental stimuli, and its main effect (β = 0.32, *p* < 0.05) is substantially stronger than the still-accumulating environmental exposures (3). Environmental factors may exert their influence indirectly through interactions with familial background, and their independent associations may only become detectable during the school-age period when near-work load intensifies. The null results we observed in the preschool period are precisely a snapshot of myopia etiology at an early developmental stage, rather than a refutation of the established evidence.

In summary, the null findings of this study should not be misinterpreted as questioning the value of environmental interventions. Rather, they should prompt us to elevate our prevention perspective from significance testing of single main effects to a systematic understanding of dynamic familial predisposition–environment interactions and developmental stage specificity. Population-wide promotion of outdoor activity and optimization of near-work behaviors should remain the cornerstone of primary prevention.

At the same time, our descriptive data reveal an alarming reality: approximately 45% of children used electronic devices at a distance less than one arm’s length, and another 35–40% at approximately one arm’s length, meaning that over 80% of preschool children failed to meet the generally recommended safe viewing distance of more than one arm’s length (approximately 30–40 cm) (4). The distribution of this adverse visual behavior was highly consistent between urban and rural children (Table 4), with no significant regional differences in screen time or outdoor activity duration (Table 3). This convergence in measured visual behaviors across urban and rural areas resonates with the convergence in myopia detection rates and warrants high priority in public health interventions (24).

### 4.4 Public health implications and policy basis

Although the present study did not detect significant independent associations of environmental behavioral factors, previous evidence indicates that early screen exposure increases the risk of myopia, particularly in children with myopic parents. The Longhua Child Cohort Study, involving nearly 26,000 preschool children, reported that early screen exposure significantly increased the risk of myopia (fixed-screen devices: OR = 2.66; mobile screen devices: OR = 2.76). Moreover, among children with myopic parents, a synergistic interaction was observed between screen exposure and insufficient outdoor activity (interaction ORs: 3.34 and 3.04, respectively) (35, 36). These findings complement our conclusion that parental myopia is the core risk factor for myopia in preschool children, suggesting that familial predisposition may amplify environmental risks through predisposition–environment interactions.

Meanwhile, data from the 2026 special survey on myopia among primary and secondary school students in Zhejiang Province, released by the Chinese Center for Disease Control and Prevention (37), also indicated that insufficient outdoor light exposure and excessive electronic screen exposure remain major modifiable factors in myopia development. Collectively, this evidence consistently supports the clear necessity of advancing the prevention threshold to the preschool stage and implementing intensified behavioral guidance for families with myopic parents.

At the policy level, the White Paper on Eye Health in China, issued by the National Health Commission in 2020, explicitly noted that myopia among children and adolescents in China is showing a trend of earlier age of onset. The Guidelines for Learning and Development of Children Aged 3–6 Years (38) likewise emphasizes the importance of vision protection in preschool children. The period from 3 to 6 years of age represents a sensitive window for visual system development, during which systematic routine vision screening, regular refractive status monitoring, and the establishment of a refractive development record for each child should be implemented. This measure serves not only as the cornerstone of a public health myopia prevention and control system (11), but is also highly consistent with the series of expert consensus statements issued by the IMI.

### 4.5 Innovations and significance

Based on the above analyses, the core innovations and academic contributions of this study can be summarized in the following three aspects.

First, uniqueness of population and methodology. Previous studies on myopia in preschool children have largely focused on either urban or rural populations in isolation. This study targeted preschool children in urban–rural fringe areas—a special population—and systematically characterized the distribution of familial predisposition in this group. Logistic regression was used to identify independent risk factors, and SEM served as a complementary tool to examine the pathway structure. The complementary use of these two methods enabled a more comprehensive examination of the pathway structure underlying the observed associations.

Second, core findings and theoretical hypothesis. We found that bilateral parental myopia is the sole independent risk factor for myopia in preschool children (OR = 4.89), and observed a numerical difference in subgroup ORs between urban (8.22) and rural (4.40) children. However, given the non-significant formal interaction test (*p* = 0.159), this difference should be interpreted as a descriptive finding rather than as evidence of effect modification. We propose the hypothesis that “familial risk becomes more prominent after convergence in measured visual behaviors,” which provides empirical support for the familial predisposition–environment interaction framework while also highlighting the need for future studies to formally test this hypothesis with adequate statistical power.

Third, practical translational value. We propose integrating “familial risk stratification” into a three-tier prevention framework for preschool myopia prevention and control, thereby offering an actionable pathway for policy formulation.

### 4.6 Limitations

Several limitations of this study should be acknowledged when interpreting the results. First, regarding the study design, its cross-sectional nature precluded the establishment of temporal and causal relationships, while the absence of dynamic retinoscopy before cycloplegia limited the identification of children with accommodative overactivity who may have been in a premyopic state. Such children would have been misclassified as emmetropic based on cycloplegic refraction alone, potentially attenuating the observed associations between near-work load and myopia. Future studies should incorporate pre-cycloplegic dynamic retinoscopy or objective accommodative function assessments to capture the full spectrum of refractive development. Second, with respect to exposure measurement, key variables, including visual behaviors and outdoor activity time, were assessed using retrospective parent-reported questionnaires and were therefore susceptible to recall bias, particularly the underestimation of screen time, and social desirability bias, without validation against objective measures such as light sensors or wearable devices. This non-differential misclassification may have biased effect estimates toward the null, potentially obscuring true associations. Therefore, the null findings for behavioral factors should be interpreted with caution and should not negate the established protective role of outdoor activity or the risk associated with excessive near work documented in randomized controlled trials and prospective cohort studies. Third, regarding data completeness, all core variables included in the analyses, including parental myopia status, screen time, viewing distance, and outdoor activity time, were complete, with no missing data and a 100% response rate. However, other non-core variables in the original questionnaire (e.g., sleep duration, dietary habits, ball sports participation, extracurricular class attendance, etc.) had small amounts of missing data and were not included in the models. Consequently, this study could not assess the potential associations of these factors on refractive status in preschool children. Future studies should incorporate objective and comprehensive environmental exposure assessments to better differentiate the contributions of familial and environmental factors. Fourth, regarding statistical analysis, the initial logistic regression did not account for the clustered sampling design, with children nested within 11 kindergartens, potentially resulting in underestimated standard errors and overly narrow confidence intervals. In addition, the limited number of myopic events (*n* = 44) relative to the number of covariates raised concerns regarding model stability and sparse-event bias. To address these issues, we performed sensitivity analyses using mixed-effects logistic regression (kindergarten as random intercept), GEE (kindergarten as clustering variable), Firth penalized regression, and L2-ridge regression, all of which indicated that parental myopia remained the only statistically significant independent risk factor, with effect estimates consistent with ordinary logistic regression (Table 6, Supplementary Table S1). Nevertheless, the limited sample size precluded more detailed subgroup or interaction analyses; future larger-scale studies are warranted to replicate these findings. Fifth, regarding conceptual definition, the observed “urban–rural convergence” was restricted to the core visual behavior indicators assessed by the questionnaire, namely screen time, viewing distance, and outdoor activity, and should not be interpreted as comprehensive equivalence in all environmental exposures between urban and rural areas, such as educational intensity, reading load, socioeconomic status, indoor light exposure, tutoring activities, and the neighborhood built environment. Moreover, axial length was not measured in this study, which prevented the assessment of structural changes underlying refractive development. Therefore, our conclusions are limited to cross-sectional “refractive status” rather than “refractive development” or “progression.” Longitudinal measurements of both refraction and axial length are needed to fully characterize the trajectory of myopia development. Sixth, confounding control. This study could not distinguish between the relative contributions of familial transmission and shared family environment to the observed parental myopia association. Future prospective studies are needed to further address this issue.

## 5 Conclusions and recommendations

### 5.1 Main conclusions

Based on this cross-sectional survey of 1,142 preschool children aged 3–6 years in Shunyi District, Beijing, the following main conclusions can be drawn: First, the overall myopia prevalence was 3.85% (44/1,142), 3.23% for urban participants and 4.39% for rural participants, with no statistically significant difference between urban and rural areas (*p* = 0.253). No significant differences were observed across sex or age groups.

Second, after adjustment for sex, age, urban–rural residence, visual behaviors, and outdoor activity time, multivariable logistic regression analysis identified myopia in both parents as the sole independent risk factor for myopia among preschool children (OR = 4.892, 95% CI: 2.675–8.954; *p* < 0.001). The association was numerically stronger in urban children (OR = 8.22) than in rural children (OR = 4.40).

Thirdly, to further examine the hypothesized pathway structure, structural equation modeling was performed as a complementary analysis. The results indicated that parental myopia showed a significant direct association with childhood myopia (standardized path coefficient *β* = 0.32, *p* < 0.05). In contrast, the direct associations of near-work load (*β* = 0.08) and outdoor activity time (*β* = −0.07) did not reach statistical significance (both *p* > 0.05). These findings indicate that familial predisposition plays a predominant role in determining refractive status in this preschool cohort, whereas cross-sectional associations of behavioral factors were not detectable in the present analysis.

Lastly, the higher prevalence of myopia among preschool children in Shunyi District than in comparable regions of China, such as Changsha and Foshan, together with the predominant role of parental myopia, highlights the need for targeted early screening and preventive interventions among children with a family history of myopia. We recommend the implementation of a multi-level evidence-based intervention strategy: primary prevention focusing on population-wide outdoor activity promotion and establishment of refractive development records; secondary prevention targeting high-risk children with myopic parents through early screening (every 6–12 months) and family-based visual behavior guidance; and tertiary prevention providing standardized correction and progression monitoring for children with established myopia.

### 5.2 Practical recommendations

Based on the core findings of this study and in alignment with domestic and international prevention guidelines, we propose a multi-level, synergistic evidence-based intervention framework targeting three tiers: the general population, high-risk individuals, and those already affected.

First, establish and standardize a refractive development record system: Develop an electronic refractive development record for all preschool children aged 3–6 years across the district, with annual cycloplegic refraction and axial length measurements (4, 11), enabling dynamic monitoring and data integration. Promote outdoor activities in kindergartens: Ensure a cumulative daily outdoor activity time of no less than 2 h (39), and incorporate this indicator into the routine kindergarten evaluation system. Second, implement risk-stratified management: For children with high-risk familial predisposition (e.g., both parents myopic), initiate an enhanced monitoring program, including refraction and axial length assessments every 6 months (11). Thirdly, standardized management of myopic children: Adopt IMI-endorsed evidence-based interventions (e.g., low-concentration atropine, defocus-based optical correction) to slow progression (10), while also attending to mental health considerations. Lastly, cross-sectoral collaboration and policy support: Integrate myopia prevention and control into public health performance assessment, establish an integrated mechanism involving healthcare, education, and family sectors, and strengthen the training of kindergarten health personnel (39).

## Data Availability

The original contributions presented in the study are included in the article/Supplementary material, further inquiries can be directed to the corresponding author.

## References

[1] Holden BA Fricke TR Wilson DA Jong M Naidoo KS Sankaridurg P . Global prevalence of myopia and high myopia and temporal trends from 2000 through 2050. Ophthalmology. (2016) 123:1036–42. doi: 10.1016/j.ophtha.2016.01.006, 26875007

[2] Long Q Tang XR Wu JJ. Investigation and analysis of myopia status and influencing factors in preschool children. Nurs Pract Res. (2022) 19:321–4. doi: 10.3969/j.issn.1672-9676.2022.03.010

[3] Lanca C Saw SM. The association between digital screen time and myopia: a systematic review. Ophthalmic Physiol Opt. (2020) 40:216–29. doi: 10.1111/opo.12657, 31943280

[4] Chu RY Qu XM. Establishing children’s refractive development record is the basic step for myopia prevention. Chin J Ophthalmol. (2009) 45:577–9. doi: 10.3760/cma.j.issn.0412-4081.2009.07.001 19957682

[5] Guo Y Liu LJ Xu L Lv YY Tang P Feng Y . Outdoor activity and myopia among primary students in rural and urban regions of Beijing. Ophthalmology. (2013) 120:277–83. doi: 10.1016/j.ophtha.2012.07.086, 23098368

[6] Qiu XY Liang SZ Yu FC. Analysis of risk factors for myopia in preschool children and discussion of intervention measures. Hainan Med J. (2016) 27:450–2. doi: 10.3969/j.issn.1003-6350.2016.03.057

[7] He M Xiang F Zeng Y Mai J Chen Q Zhang J . Effect of time spent outdoors at school on the development of myopia among children in China: a randomized clinical trial. JAMA. (2015) 314:1142–8. doi: 10.1001/jama.2015.10803, 26372583

[8] Jones-Jordan LA Sinnott LT Graham ND Cotter SA Kleinstein RN Manny RE . The contributions of near work and outdoor activity to the correlation between siblings in the collaborative longitudinal evaluation of ethnicity and refractive error (CLEERE) study. Invest Ophthalmol Vis Sci. (2014) 55:6333–9. doi: 10.1167/iovs.14-14640, 25205866 PMC4193758

[9] Rudnicka AR Kapetanakis VV Wathern AK Logan NS Gilmartin B Whincup PH . Global variations and time trends in the prevalence of childhood myopia: a systematic review and meta-analysis. Br J Ophthalmol. (2016) 100:882–90. doi: 10.1136/bjophthalmol-2015-307724 PMC4941141 26802174

[10] International Myopia Institute. IMI—defining and classifying myopia: a proposed set of standards for clinical and epidemiologic studies. Invest Ophthalmol Vis Sci. (2019) 60:M20–30. doi: 10.1167/iovs.18-25957, 30817826 PMC6735818

[11] International Myopia Institute. IMI—clinical management guidelines report. Invest Ophthalmol Vis Sci. (2019) 60:M184–203. doi: 10.1167/iovs.18-25977, 30817832

[12] Chua SYL Sabanayagam C Cheung YB Chia A Valenzuela RK Tan D . Age of onset of myopia predicts risk of high myopia in later childhood in myopic Singapore children. Ophthalmic Physiol Opt. (2016) 36:388–94. doi: 10.1111/opo.12305, 27350183

[13] Kim JM Choi YJ. Nutritional intake, environmental factors, and their impact on myopia prevalence in Korean children aged 5–12 years. J Health Popul Nutr. (2024) 43:14. doi: 10.1186/s41043-024-00506-6, 38287408 PMC10823653

[14] Wang CY Hsu NW Yang YC Chen YL Shyong MP Tsai DC. Premyopia at preschool age: population-based evidence of prevalence and risk factors from a serial survey in Taiwan. Ophthalmology. (2022) 129:880–9. doi: 10.1016/j.ophtha.2022.03.017, 35331752

[15] Grzybowski A Kanclerz P Tsubota K Lanca C Saw SM. A review on the epidemiology of myopia in school children worldwide. BMC Ophthalmol. (2020) 20:27. doi: 10.1186/s12886-019-1220-0, 31937276 PMC6961361

[16] Ophthalmology Branch of the Chinese Medical Association, Strabismus and Pediatric Ophthalmology Group. Chinese expert consensus on myopia prevention and control in preschool children (2023 edition). Chin J Ophthalmol. (2023) 59:721–7.

[17] He X Sankaridurg P Wang J Chen J Naduvilath T He M . Time outdoors in reducing myopia: a school-based cluster randomized trial with objective monitoring of outdoor time and light intensity. Ophthalmology. (2022) 129:1245–54. doi: 10.1016/j.ophtha.2022.06.024, 35779695

[18] Liao SX Li XY Bai N Wu DP Yang WL Wang F . An empirical study on the effect of outdoor illumination and exercise intervention on children’s vision. Front Public Health. (2023) 11:1270826. doi: 10.3389/fpubh.2023.1270826, 38155899 PMC10754518

[19] Xie XL Zhu BD Li SJ. Analysis of vision and refractive status in preschool children. Int Eye Sci. (2023) 23:132–5. doi: 10.3980/j.issn.1672-5123.2023.1.34

[20] Rose KA Morgan IG Ip J Kifley A Huynh S Smith W . Outdoor activity reduces the prevalence of myopia in children. Ophthalmology. (2008) 115:1279–85. doi: 10.1016/j.ophtha.2007.12.019, 18294691

[21] Morgan IG French AN Ashby RS Guo X Ding X He M . The epidemics of myopia: aetiology and prevention. Prog Retin Eye Res. (2018) 62:134–49. doi: 10.1016/j.preteyeres.2017.09.004, 28951126

[22] Wu PC Chen CT Lin KK Sun CC Kuo CN Huang HM . Myopia prevention and outdoor light intensity in a school-based cluster randomized trial. Ophthalmology. (2018) 125:1239–50. doi: 10.1016/j.ophtha.2017.12.011, 29371008

[23] Li F Zhang TH Qu KC Zhang JL Zhu YZ Yu QY . A study on urban–rural differences in myopia and its influencing factors among preschool children in Wuhan. J Guangxi Med Univ. (2026) 43:273–82. doi: 10.16190/j.cnki.45-1211/r.2026.02.015

[24] Verhoeven VJM Morgan IG Guggenheim JA. Myopia is predominantly genetic or predominantly environmental? Ophthalmic Physiol Opt. (2025) 45:911–7. doi: 10.1111/opo.13464, 40028922 PMC12087860

[25] Lin Z Vasudevan B Ciuffreda KJ Gao TY Zhou HJ Liang YB. Generational difference of axial length and its risk factors in urban and rural China. J Ophthalmol. (2019) 2019:1607064. doi: 10.1155/2019/1607064, 31885881 PMC6899268

[26] Zong CX Hu JP Jia HY . Meta-analysis of the effect of parental myopia on children’s myopia. Chin J Ophthalmol. (2024) 33:368–76. doi: 10.13281/j.cnki.issn.1004-4469.2024.05.008

[27] Ying ZQ Li DL Zheng XY Zhang XF Pan CW. Risk factors for myopia among children and adolescents: an umbrella review of published meta-analyses and systematic reviews. Br J Ophthalmol. (2024) 108:167–74. doi: 10.1136/bjo-2022-322773, 36754586

[28] Pu J Fang Y Zhou Z Chen W Hu J Jin S . The impact of parental myopia and high myopia on the hyperopia reserve of preschool children. Ophthalmic Res. (2024) 67:115–24. doi: 10.1159/000535193, 37989114

[29] Wang Y Zhu H Zhu GW Pan H Qian DJ Li J . Factors associated with hyperopia reserve among preschoolers in Suzhou high-tech zone. Chin J Sch Health. (2023) 44:599–601. doi: 10.16835/j.cnki.1000-9817.2023.04.028

[30] Ma ZF Hou J Mi XJ Chen W Pu JN Jiao YH . Investigation of myopia prevalence and influencing factors in preschool children in Beijing Haidian district. Ophthalmol China. (2024) 33:362–6. doi: 10.13281/j.cnki.issn.1004-4469.2024.05.007

[31] Chen CW Yao JY. Evaluation of risk factors for childhood myopia progression: a systematic review of the literature. Indian J Ophthalmol. (2024) 72:S721–7. doi: 10.4103/IJO.IJO_1909_23, 39141497 PMC11670848

[32] Wang MM Zhu LL. Chain analysis of influencing factors of myopia in children and adolescents based on structural equation model and the mediating role of behavior and environment. J Front Med. (2025) 15:122–6. doi: 10.20235/j.issn.2095-1752.2025.32.029

[33] Morgan IG Wu PC Ostrin LA Tideman JWL Yam JC Lan W . IMI risk factors for myopia. Invest Ophthalmol Vis Sci. (2021) 62:3. doi: 10.1167/iovs.62.5.3, 33909035 PMC8083079

[34] International Myopia Institute. IMI 2025 digest. Invest Ophthalmol Vis Sci. (2025) 66:M1–M50. doi: 10.1167/iovs.66.3.M1

[35] Yang GY Huang LH Schmid KL Li CG Chen JY He GH . Associations between screen exposure in early life and myopia amongst Chinese preschoolers. Int J Environ Res Public Health. (2020) 17:1056. doi: 10.3390/ijerph17031056, 32046062 PMC7037286

[36] Huang L Schmid KL Yin XN Zhang J Wu J Yang G . Combination effect of outdoor activity and screen exposure on risk of preschool myopia: findings from Longhua child cohort study. Front Public Health. (2021) 9:607911. doi: 10.3389/fpubh.2021.607911, 33748061 PMC7973103

[37] Liu Q Liu CY Shi YY Wei XY Chen F Wei YZ . Prevalence and influencing factors of myopia among primary and secondary school students—Zhejiang province, China, 2023. China CDC Wkly. (2026) 8:154–60. doi: 10.46234/ccdcw2026.023, 41953469 PMC13054702

[38] Ministry of Education. Guidelines for the Development and Learning of Children Aged 3–6 Years. Beijing: Ministry of Education (2012).

[39] National Health Commission, National Disease Control and Prevention Administration, Cyberspace Administration of China, National Development and Reform Commission, Ministry of Education, Ministry of Finance, (2025) Notice on issuing the action plan for promoting the “five health” dimensions among children and adolescents (2026–2030): Guo Wei Fu fa [2025] no. 21 [EB/OL]. Available online at: https://www.gov.cn/zhengce/zhengceku/202512/content_7053270.htm (Accessed August 2, 2026)

