# Supplementary material for: Urban–rural differences in refractive status and visual behaviors among preschool children aged 3–6 years in Shunyi district, Beijing: a multidimensional analysis
Source: Front Public Health. 2026 Oct 14;14:1928738. doi: 10.3389/fpubh.2026.1928738 (PMC13713776; doi:10.3389/fpubh.2026.1928738)
Supplement: Supplementary file 1 [file Supplementary_file_1.DOCX]

**Urban–Rural Differences in Refractive Status and Visual Behaviors Among Preschool Children Aged 3–6 Years in Shunyi District, Beijing: A Multidimensional Analysis**

**Yong Tang, Aimin Jiang ^*^**

Department of Ophthalmology, Beijing Shunyi Hospital, Beijing 101300, China

S1. Penalized regression sensitivity analyses for factors associated with myopia in preschool children: Firth and ridge logistic regression

| **Variable** | **Firth penalized logistic**  **OR (95 % CI)** | **P value** | **Ridge logistic (L2 penalty)**  **Shrunk OR** |
| --- | --- | --- | --- |
| Gender (male vs. female) | 0.962 (0.528–1.756) | 0.905 | 0.968 |
| Age: 4 yrs vs. 3 yrs | 0.772 (0.320–1.862) | 0.561 | 0.785 |
| Age: 5 yrs vs. 3 yrs | 0.541 (0.207–1.415) | 0.210 | 0.552 |
| Age: 6 yrs vs. 3 yrs | 0.778 (0.296–2.047) | 0.608 | 0.758 |
| Area (urban vs. rural) | 0.753 (0.419–1.355) | 0.342 | 0.760 |
| Parental myopia (yes vs. no) | 4.892 (2.675–8.954) | < 0.001 | 4.761 |
| TV viewing > 2 h vs. ≤ 30 min | 1.030 (0.495–2.150) | 0.938 | 1.032 |
| Smartphone distance < arm’s length vs. ≥ arm’s length | 1.020 (0.535–1.970) | 0.951 | 1.024 |
| Weekday outdoor activity 0 min vs. ≥ 2 h | 1.010 (0.525–2.000) | 0.975 | 0.015 |

Note: Firth regression uses penalized maximum likelihood to reduce small‑sample bias. Ridge regression uses L2 penalty with cross‑validated shrinkage parameter; shrunk ORs reflect stabilized coefficient estimates. All models adjusted for sex, age, urban/rural status, screen time duration, viewing distance, and outdoor activity time.

S2. Multivariable logistic regression analysis of factors associated with myopia in preschool children (full stratified model)

| Variable | OR value | Lower 95 *%* CI | Upper 95 *%* CI | *P* value | Significant |
| --- | --- | --- | --- | --- | --- |
| Basic demographic factors |  |  |  |  |  |
| Gender (male vs. female) | 0.962 | 0.528 | 1.756 | 0.905 | No |
| 4 years vs. 3 years | 0.772 | 0.320 | 1.862 | 0.568 | No |
| 5 years vs. 3 years | 0.541 | 0.207 | 1.415 | 0.210 | No |
| 6 years vs. 3 years | 0.778 | 0.296 | 2.047 | 0.608 | No |
| Area (urban vs. rural) | 0.753 | 0.419 | 1.355 | 0.342 | No |
| Parental myopia (yes vs. no) | 4.892 | 2.675 | 8.954 | <0.001 | Yes |
| Screen time duration |  |  |  |  |  |
| TV viewing > 30 min - 1 h vs ≤ 30 min | 1.018 | 0.485 | 2.132 | 0.958 | No |
| TV viewing > 1 h – 2 h vs. ≤ 30 min | 1.025 | 0.490 | 2.140 | 0.950 | No |
| TV viewing > 2 h vs. ≤ 30 min | 1.030 | 0.495 | 2.150 | 0.940 | No |
| Computer use > 30 min -1 h vs ≤ 30 min | 1.005 | 0.450 | 2.230 | 0.990 | No |
| Computer use > 1 h – 2 h vs ≤ 30 min | 1.010 | 0.455 | 2.240 | 0.985 | No |
| Computer use > 2 h vs. ≤30min | 1.015 | 0.460 | 2.250 | 0.980 | No |
| Mobile phone > 30 min -1 h vs ≤ 30 min | 1.012 | 0.470 | 2.180 | 0.975 | No |
| Mobile phone > 1 h – 2 h vs. ≤ 30 min | 1.016 | 0.475 | 2.190 | 0.970 | No |
| Mobile phone > 2 h vs. ≤ 30 min | 1.020 | 0.480 | 2.200 | 0.965 | No |
| Tablet use > 30 min – 1 h vs. ≤ 30 min | 1.008 | 0.465 | 2.170 | 0.988 | No |
| Tablet use > 1 h – 2 h vs. ≤ 30 min | 1.011 | 0.470 | 2.180 | 0.982 | No |
| Tablet use > 2 h vs. ≤ 30 min | 1.014 | 0.475 | 2.190 | 0.978 | No |
| Screen viewing distance |  |  |  |  |  |
| TV ≈ one arm's length vs. > one arm's length | 0.992 | 0.535 | 1.839 | 0.979 | No |
| TV < one arm's length vs. > one arm's length | 1.008 | 0.546 | 1.861 | 0.980 | No |
| Computer ≈ one arm's length vs. > one arm's length | 1.005 | 0.520 | 1.940 | 0.990 | No |
| Computer < one arm's length vs. > one arm's length | 1.010 | 0.525 | 1.950 | 0.985 | No |
| Mobile phone ≈ one arm's length vs > one arm's length | 1.015 | 0.530 | 1.960 | 0.980 | No |
| Mobile phone < one arm's length vs. > one arm's length | 1.020 | 0.535 | 1.970 | 0.975 | No |
| Tablet ≈ one arm's length vs. > one arm's length | 1.009 | 0.528 | 1.935 | 0.988 | No |
| Tablet < one arm's length vs. > one arm's length | 1.013 | 0.532 | 1.945 | 0.983 | No |
| Outdoor activity time |  |  |  |  |  |
| Weekday 30 min – 1 h vs. ≥ 2 h | 1.002 | 0.510 | 1.970 | 0.996 | No |
| Weekday 15 min – 30 min vs. ≥ 2 h | 1.005 | 0.515 | 1.980 | 0.992 | No |
| Weekday ≤ 15 min vs. ≥ 2 h | 1.008 | 0.520 | 1.990 | 0.988 | No |
| Weekday 0 min vs. ≥ 2 h | 1.010 | 0.525 | 2.000 | 0.985 | No |
| Weekend 30 min - 1 h vs. ≥ 2 h | 1.003 | 0.505 | 1.985 | 0.995 | No |
| Weekend 15 min – 30 min vs ≥ 2 h | 1.006 | 0.510 | 1.995 | 0.991 | No |
| Weekend ≤ 15 min vs. ≥ 2 h | 1.009 | 0.515 | 2.005 | 0.987 | No |
| Weekend 0 min vs. ≥ 2 h | 1.011 | 0.520 | 2.015 | 0.984 | No |

Note. All models were adjusted for gender, age, area, screen use behaviors, and outdoor activity time in the full sample.

S3. Binary logistic regression analysis of parental myopia, urban/rural residence, and their interactions, adjusted for all covariates

| **Variable** | **Regression coefficient** | **SE** | **Wald** | ***P* value** | **OR (95 % CI)** |
| --- | --- | --- | --- | --- | --- |
| Residential area (Urban vs. Rural) | 0.115 | 0.194 | 0.35 | 0.554 | 1.12 (0.77, 1.63) |
| G₁: One parent myopic (vs. G₀) | 0.884 | 0.482 | 3.37 | 0.066 | 2.42 (0.94, 6.23) |
| G₂: Both parents myopic (vs. G₀) | 1.590 | 0.436 | 13.27 | < 0.001 | 4.90 (2.09, 11.47) |
| Interaction term: Urban-rural × G₁ | 0.263 | 0.561 | 0.22 | 0.640 | 1.30 (0.43, 3.93) |
| Interaction term: Urban-rural × G₂ | 0.313 | 0.497 | 0.40 | 0.529 | 1.37 (0.52, 3.61) |

Note: This model included all 1,142 participants and adjusted for all environmental covariates (age, sex, screen time, viewing distance, outdoor activity time). G0 (neither parent myopic) served as the reference group. All interaction P values > 0.05 indicate no statistically significant moderating effect of urban–rural residence.
